# Schuylkill Valley Veterinary Association

**Published:** 1901-01

**Authors:** E. D. Longacre

**Affiliations:** Secretary


### SCHUYLKILL VALLEY VETERINARY ASSOCIATION.

THE semi-annual meeting of this Association was held at the Board of Trade Rooms, Reading, Pa., December 19, 1900. The meeting was called to order by the President, Dr. Otto Noack. The members present were: Drs. Otto Noack, Reading; I. C. Newhard, Ashland; U. S. G. Bieber, Kutztown; E. D. Longacre, Shenandoah; F. H. Schneider, Philadelphia; D. R. Kohler, Boyertown; W. S. Longacre, Mantz; G. H. Wehr, Denver; W. G. Huyett, Wernersville. The President made the opening address, which was much appreciated by the members present. Business of the different committees was transacted and the following resolutions passed:

*Whereas*, The registration of veterinarians with the prothonotaries of the counties throughout the State shows so many violations of the laws, voluntary and involuntary; therefore, be it

*Resolved*, That the Legislature be appealed to leave the registration of veterinarians entirely with the State Board of Veterinary Medical Examiners, which is created in the interests of the people of the State.

*Whereas*, Comparative medicine has become such a potent factor in public life, and its study, with the daily new developments, so voluminous that its branches can only be fully understood by thoroughly educated people; therefore, be it

*Resolved*, That the dean and faculty of the veterinary colleges, especially those of the University of Pennsylvania, should endeavor to elevate the standard for matriculation and extend the course of study to four years.

U. S. G. Bieber, of Kutztown, was a delegate to the American Veterinary Medical Association at Detroit, Mich., and reported the proceedings of the meeting, which was very interesting.

Papers were read by Dr. I. C. Newhard on "Mange" and Dr. D. R. Kohler on "Tetanus." Both papers were well prepared and a lively discussion followed, both spirited and interesting. Drs. W. S. Longacre, G. H. Wehr, Otto Noack, and E. D. Longacre spoke of navicular trouble and discussed it to its full extent.

Meeting adjourned to meet at Pottsville at the usual time in 1901.

E. D. Longacre, V.S.,
Secretary.

